# Peddling promise? An analysis of private umbilical cord blood banking company websites in Canada

**DOI:** 10.1007/s10561-021-09919-7

**Published:** 2021-04-23

**Authors:** Alessandro R. Marcon, Blake Murdoch, Timothy Caulfield

**Affiliations:** Faculty of Law, Health Law Institute, University of Alberta, Edmonton, Canada

**Keywords:** Cord blood, Commercialization, Marketing, Public perceptions, Biotechnology

## Abstract

Private umbilical cord blood banking is growing around the world. A family’s decision to bank cord blood publicly or privately can be influenced by numerous sources including healthcare practitioners, personal networks, the popular press, social media and marketing discourse from private entities. Issues have been raised concerning how private banks market their services, particularly with regards to the likelihood of use and for what purposes cord blood can be used. The objective of this study was to analyze the marketing on the seven company websites offering private cord blood storage in Canada. We performed a mix of content and general qualitative analysis on the seven websites. Our analysis shows substantial hype around cord blood uses, amplifying the promise of speculative uses and distorting the likelihood of use. Findings show that this promotional messaging often deploys communication strategies which draw on testimonials and emotionally-charged narratives. Questions should be asked about whether the promissory aspects of these websites constitute breaches of Canadian law or regulation. Careful monitoring of the private cord blood space is important for ensuring that the Canadian public is adequately and accurately informed of the services being offered.

## Introduction

### Context

Umbilical cord blood banking has grown significantly over the past two decades. The option was first made available in the 1990s following the discovery that cord blood is a rich source of stem cells (Kurtzberg 2017). These stem cells can now be used to treat blood and immune system disorders, and also in research on novel therapies not yet ready for clinical application (Allan 2020; W. T. Shearer et al. 2017). Unlike the United Kingdom, which has had an operational public banking program since 1996 (Haw 2016), and even though Quebec established a functioning provincial system in 2004, it was not until 2013 that Canada developed a national public banking program (Haw 2016; Héma-Québec 2012; NHS, access 2020). Private cord blood banks began offering storage in Canada as early as 1996 (Haw 2016) and have expanded significantly since. The current global market is projected to be worth $23 billion by 2025 (Global News Wire 2019).

Public banking is overseen by government agencies, free of cost for the donor, and the collected materials are used to treat other patients or for research. Collecting as much cord blood as possible from a wide variety of ethnic groups is important to ensure opportunities for Canada’s ethnically diverse population. Private banking is for-profit, and stores cord blood for clients until retrieval is requested. Individuals generally pay an initial fee (approximately $1000 CAD), which covers registration and collection, followed by a yearly storage fee (approximately $125). There are currently seven private banking companies marketing in Canada (Parent’s Guide to Cord Blood 2020). Since both public and private institutions are interested in obtaining cord blood, Canadian parents are tasked with obtaining accurate, up-to-date information about whether and where to donate or store the cord blood following childbirth.

Decisions to bank publicly or privately can be influenced by numerous sources including healthcare practitioners, personal networks, the popular press, social media and marketing discourse (Graham et al. 2016; Morgan et al. 2005; Peberdy et al. 2018; Soroka et al. 2013). Popular health websites such as www.babycenter.ca or www.webmd.com have pages dedicated to explaining the differences between public versus private banking (Private cord blood banking in Canada access 2020; Umbilical cord blood banking access 2020). Research has shown, however, the public’s awareness and knowledge of cord blood uses is lacking, and that information sources accessed by the public can be “varied, fragmented and inconsistent” (Peberdy et al. 2018). The differing and often opposing discourses produced by public and private institutions likely contribute to this confusion.

Tensions exist in Canada between private and public cord blood banking. In Canada, as well as abroad, policy statements and government discourse recommend public banking over private, except in cases where a family might benefit from cord blood use based on an established prior condition like, for example, a sibling with leukemia, sickle cell disease, Hodgkin’s lymphoma or thalassemia (MyHealth Alberta access 2020; Health Link BC access 2020; Health Canada 2019; Shearer et al. 2017). These recommendations have been made because the potential for autologous (for donor) or allogeneic (for others) use offered by private banking is extremely low–a point confirmed by usage data (Shearer et al. 2017). Private banking is also costly and can elude regulatory oversight, in turn impacting overall blood quality (Shearer et al. 2017). In contrast, public banks provide public health benefits and regulatory oversight, which helps to ensure effectiveness while minimizing risk (Mohammed and EL Sayed 2015; MyHealth Alberta access 2020; HealthLink BC, access 2020; Health Canada 2019). Provincial and federal governments have also stressed how private banks commonly oversell the potential for cord blood use, especially with regards to more speculative therapies where evidence to support clinical application is lacking (Mohammed and EL Sayed 2015; MyHealth Alberta access 2020; HealthLink BC, access 2020; Health Canada 2019).

Academic research investigating the public–private divide has described how public banks typically operate on a “regime of truth,” in contrast to private banks which typically operate on a “regime of promise” (Brown 2013; Martin et al. 2008). Here, private banks often apply the element of hope to their marketing discourse, upselling potential benefits, and thus playing into the “promissory” (Petersen and Krisjansen 2015) and “hyping” (T. Caulfield and Condit 2012; Master and Resnik 2013; Montague 2019) elements of the health sciences. Private banking has also been marketed as type of “biological insurance” for parents wanting to do whatever is possible for their children’s and family’s benefit (Brown 2013; Marcon et al. 2020; Martin et al. 2008; Weeks 2018). Indeed, a labeling shift has been observed with private banks now typically describing their services as “family banks” (Brown 2013; Martin et al. 2008; Weeks 2018). Further research documenting the procedures undertaken by women who stored blood privately in Canada found the process to be more complex and arduous than the women had expected (Haw 2016). Notably, a range of critical commentary also exists around public banking processes (Allan et al. 2016; Brown 2013; Isasi et al. 2013, 2018). In some contexts, critiques of drawing absolute distinctions between public and private banks have been raised, noting that similar marketing efforts are present in both (Beltrame 2020).

The banking of cord blood will likely continue to increase both publicly and privately. Indeed, recent research on the portrayal of cord blood in the North American popular press has found that the topics of public and private banking feature significantly (Marcon et al. 2020). This research also shows that while private banking was more commonly portrayed as problematic than beneficial, strong and potentially persuasive narrative messaging around private banking benefits was also present, which might impact a family’s decision (Caulfield et al. 2019; Marcon et al. 2020). The public is increasingly going online to access health information (Shearer and Gottfried 2017), and because both the public’s knowledge and awareness of cord blood uses is lacking and because private cord blood banks typically oversell the potential for cord blood use, it is vital to observe and analyze the information influencing cord blood banking decisions. This includes analyzing the online marketing of the private cord blood banks as these companies’ practices may elude regulatory oversight, particularly with regards to misleading the public around the probability of usefulness and the practical benefits of private cord blood banking. Indeed, these companies require accreditation to operate in Canada, and it is their responsibility to adhere to government mandated truth-in-advertising standards (Murdoch et al. 2020).

### Objective

The objective of this research was to analyze the marketing on the seven cord blood companies’ websites offering services in Canada. While research on the marketing of cord blood exists for other contexts or periods (i.e., Beltrame 2020; Brown 2013), there is no research for the current Canadian context. Given the concerns raised around private-banking marketing, we considered it valuable to analyze the manner in which the companies are marketing their services to the public. We sought to analyze specific characteristics and themes evident on the websites, and the degree to which websites promoted the growing future potential of stem cell use as a reason to store cord blood in a private bank.

## Methods

### Data collection

Of the seven private cord blood banking companies in Canada, six were based outside of Quebec and had websites in English. The company in Quebec used a website equipped with an auto-translate function. Five of the seven websites were not an extension of a parent company or website, and were therefore designed specifically for the region of Canada. Clinique OVO, based in Quebec, did not have an entire website devoted to cord blood banking but rather one webpage labelled “stem cells/bioinsurance” situated inside of their homepage “https://www.cliniqueovo.com/.” Future Health BioBank [Futurehealthbiobank.com] operates internationally and had a Canadian specific URL [https://futurehealthbiobank.com/ca-en/]. In February 2020 these six complete websites and the one webpage for Clinique OVO were cached. All analysis was of these cached versions of the websites as they appeared in February, in order to maintain data consistency throughout analysis.

### Data analysis

Analysis of the websites consisted of performing a mix of directed content analysis (Hsieh and Shannon 2005) and an overall qualitative analysis deploying a “general inductive approach” (Thomas 2003) informed by the procedures of “grounded theory” (Higginbottom and Lauridsen 2014). Central to this approach is allowing the findings to emerge out of the dominant and frequent aspects of the data made salient through numerous and repeated observations. Thus, the general context of the private cord blood environment was taken into consideration when engaging the websites, yet observations of the websites were not restricted to preconceptions or predefined criteria. The initial objective was to observe, as mentioned, how current and future uses of stem cells were portrayed on the websites. Analysis also captured any additional messaging characteristics observed across the websites.

The directed content analysis was applied to the websites’ homepages, capturing the presence and absence of central salient characteristics, all of which are listed in Table 1 of the results section. It was deemed valuable to discern which characteristics and what information companies presented as the face of the company—the first, and potentially only, online point of contact with consumers—and which elements were shared among all companies. The qualitative themes were constructed by viewing each website in its entirety and making detailed observations, and then using these observations for repeated rounds of analysis. A general summary of shared website characteristics was documented along with distinctive marketing strategies used by different companies. As a result of detailed analysis on all websites, three central themes became evident: **Cord Blood Uses**, **Storage Options**, and **Social Benefits**.

The theme of **Cord Blood Uses** included how websites detailed the current and future uses of cord blood stem cells, as well as whether and how the likelihood of use was presented. Indeed, government discourse and academic literature list the potential use of privately-banked cord blood as extremely low (Shearer et al. 2017). The theme of **Storage Options** had two central components: the portrayal (if any) of public cord blood banking and the messaging that not privately banking cord blood was wasting a resource. The theme of **Social Benefits** pertained to messaging of customer benefits outside of clinical applications. This included how banking blood privately might make one feel, or why certain kinds of individuals would choose the private banking option.

## Findings

### Homepage and general overview

#### Front page

Table 1 presents an overview of the homepage characteristics. All websites included images and, notably, all websites except one included images solely of Caucasians. Some websites had multiple language presentation options, notably French (on four) and Mandarin/Cantonese (on three). Websites provided links to social media, most notably Facebook (on all) and Twitter (on six). All social media accounts had been active over the past 6 months, although some companies appeared to be (significantly) more active on these platforms than others. All of the websites offered cord tissue storage in addition to cord blood, and one website (Future Health) also offered dental pulp storage. Six of the seven websites displayed accreditation and included a Frequently Asked Questions (FAQ) section. Six of the seven websites’ homepages highlighted numerical statistics, which, in five cases, related to the message that cord blood could be used to treat “more than” or “over” 80 diseases. Five of the seven websites provided links to free information packages or brochures and/or information sessions that can be attended. Three websites included information on speculative, regenerative treatments on their homepage. Only one website included a link to a government website, and that was Clinique OVO, which, as noted, was only a single webpage inside of a larger website. All other homepage characteristics for all websites are displayed in Table 1.

**Table 1:** Homepage attributes for the 7 Canadian private cord blood banking companies’ websites

Website homepage attribute	Cord blood banking company						
	Cells for Life	Progenics	CReATe	Healthcord	Clinique OVO	Insception	Future Health
Images	Y (video images–white only)	Y (white only)	Y (white only)	Y (white only)	Y (white family + team (2))	Y (multi-ethnic)	Y (white only)
Other languages (any presence)	Y (French)	Y (Chinese)	N	Y (French, Chinese)	Y (French default, English)	Y (Chinese)	Y (French)* 34 other countries can be chosen
Videos	Y	N	N	N	Y	Y	N
Social media	Y (4) Facebook Twitter YouTube Google +	Y (3) Facebook Twitter Google +	Y (4) Facebook Twitter Pinterest Instagram	Y (2) Facebook Twitter	Y (5) Facebook Twitter YouTube Google + LinkedIn	Y (4) Facebook Twitter YouTube LinkedIn	Y (1) Facebook* but with UK listed as base
Social media activity in 2020	N (November 2019)	Y	Y	Y	Y	Y	Y
FAQs	Y	Y	Y	Y	N	Y	Y
Blog	Y	N	Y	N	N	Y	Y
Accreditation	N	Y	Y	Y	Y* (not peristem)	Y	Y
Testimonials	N	Y (stock image − short text)	N* client profile	N	N	Y	Y
Information packages and seminars	Y	N	Y	N	Y	Y	Y
Numbers related to use	Y (treatment 80 + diseases, 40 K + transplants worldwide)	Y (treatment 80 + diseases)	N* (life-threatening diseases)	Y (treatment 80 + diseases)	Y (treatment 80 + diseases, 30 K + transplants worldwide)	Y (treatment 80 +)	Y (200 K + processed and stored, 100 K + families, “treat conditions”
Mentioning of regenerative therapy	Y	N	Y	N	N	N	Y (trials and use)
Mentioning of affordability	Y	Y	N	Y	N–only lists of costs	Y	N
Cord tissue (including Peristem (P-stem))	Y	Y	Y (P-stem)	Y	Y (P-stem)	Y	Y (+ dental pulp)
Community connections/ Partners (organizations, hospitals, etc.)	N	Y	Y	N	N	Y	N
First phrase	“Cord blood…too precious to waste”	“High consistent quality”	N/A- feature on client with successful procedure	“Canada’s trusted name in Cord Blood and Tissue Banking”	“The birth of a child is a unique opportunity to harvest the precious stem cells found in the umbilical cord blood cells”	“Expecting a baby? Request your cord blood info kit here”	4 images on rotation. First: “A once in a lifetime opportunity to protect your child’s future health.”
Links to government websites	N	N	N	N	Y	N	N
Link to clinical trials	N	N	N	N	Y	Y	Y
Links for “Health care providers”	Y	N	N	N	N	Y	N

(*Y*  yes, *N* no)

#### General overview

##### Common website characteristics

All websites contained the following five elements: (1) a presentation of what cord blood (and cord tissue) is, and how both can be used; (2) a detailing of why one should store cord blood (and cord tissue); (3) a presentation of the cord blood collection procedures, at times including elaborate details on collection kits and storage equipment; (4) a breakdown of pricing options, including promotions and payment plans; and (5) information about the company including accreditation, experience, “about us” information (e.g. “our experts,” institutional collaborators, etc.), and particular company attributes or selling points (see Table 1 for phrases present on each homepage).

Across the websites, collection and storage procedures were typically portrayed as safe and uncomplicated. However, an exception was Healthcord, which described the process as “complex” and requiring scientific expertise. Extensive detail was provided regarding collection equipment and pictures were commonly present outlining each step of procedure. Interestingly, the details around a maternal blood draw, which is required to take place between one and seven days after childbirth, were often mentioned only in footnotes or complimentary materials to download. The exceptions were Clinique OVO and Insception, which mentioned the maternal blood draw explicitly in collection procedures Several websites mentioned that tests need to be done on maternal blood but do not provide procedural details concerning the blood draw. When detailing procedures, some detail around storage lifespan was provided (i.e. “20 years” [Progenics], “25 years” [Future Health]), yet all websites offered storage plans up to and exceeding 20 years. On some websites, such as Cells for Life, CReATe, and Progenics, there was messaging that “theoretically” or “there is reason to believe” stem cells could be stored “indefinitely.” Websites typically provided statistics around “100% successful” storage and/or release rates.

All companies charged similar fees of approximately $1000 initially and $125 per year thereafter. The sole exception was Future Health, which charged approximately $1500 for initial storage. All websites included some rhetoric around affordability, and offered promotions for returning clients, multiple donors, storing for multiple years, or for storing cord tissue (or “Peristem”) in addition to cord blood.

##### Key marketing distinctions

Each website foregrounded certain aspects of their company which served to distinguish their services from others. Future Health, for example, stated on their homepage that they are the only Canadian bank to offer dental pulp stem cell storage, and described their company as “one of the world’s most accredited” banks. In addition to detailing all of their 22 accreditations, Future Health described their “unique benefits” of having the “largest global presence” and having released the “most treatment samples.” Future Health’s website also uniquely included a chart comparing their services with four other Canadian banks (Insception, Cells4life [sic], CReATe, and Clinque OVO), which highlighted Future Health’s claim to superior service.

Insception called itself “Canada’s #1 Cord Blood Program” and was the only company to include multiethnic families on its homepage. They claimed to be the only company with a “nationwide presence,” having stored three-quarters of the total cord blood stored in Canada. Insception also had a strong testimonial/family story presence both on their homepage and throughout their website. Like Insception, CreATe foregrounded a family story on their homepage, which appeared front and centre as the principal message underneath headings. Deeper in the site, CReATe stated their “technology offers the most advanced stem cell services available in the North American market.” Healthcord, like Insception, labeled themselves as “Canada’s #1 Cord Blood Bank” as well as “Canada’s Trusted Name.” Progenics describe themselves as “a leader… with over 15 years experience,” and stated that they are “the only family cord blood bank in the world that publishes their quality testings.”

Cells for Life, like Insception, included a strong presence of testimonial/family stories on their homepage and throughout the website. Additionally, Cells for Life highlighted their experience by labelling themselves as “Canada’s cord blood and tissue experts” and by stating that they have “over 20 years of experience.” Clinique OVO bioinsurance is uniquely, as previously mentioned, a single webpage inside of Clinique OVO’s parent website. A unique characteristic of their website was the foregrounding of Canadian public institutions, such as Health Canada. They described their services in which “Global requirements [are] respected” and provided links not only to Health Canada but also to Héma Québec and the Society of Obstetricians and Gynecologists of Canada.

### Complete website

#### Theme of use

##### Treatments: established (“today”) and speculative (“future”/“emerging”)

All websites provided information on how cord blood can be used, separating usage into two categories: (1) “treatable” diseases, also commonly labelled “current” uses or uses “today,” and (2) “emerging” diseases, also labelled with rhetoric like “future” use, or with terms like “potentially,” “regenerative” and in one case, “diseases in clinical trial” [Cells for Life]. Regarding established treatments, meaning those that are scientifically and clinically validated, the common messaging across all websites, with one exception, was that cord blood can be used to treat more than 80 diseases. Indeed, this message appeared on five of the seven websites’ homepages. It was also the message that appeared on the homepage of Canadian Blood Services (CBS), which reads, “Like bone marrow or peripheral blood stem cell donation, donated cord blood can also treat over 80 diseases and disorders.” (The one exception was CReATe’s website, which stated that cord blood can “treat more than 70 different life threatening diseases.”) When detailing the treatment for 80+ diseases, websites refer primarily to blood cancers (most notably leukemia), immune disorders, bone marrow failure, and a few other diseases for which evidence of efficacy exists. Often, complete lists of treatable diseases were provided. In addition to and often appearing in conjunction with the detailing of established treatment possibilities was the rhetoric of cord blood being a “perfect match” for the donor as well as of (high) potential use for other members of the family, which was usually accompanied by explanations of autologous and allogenic use.

Regarding the speculative uses of cord blood, the common message across all websites was that research and clinical trials are underway and that because of stem cells’ potential, considerable expansion in uses is expected to occur in the future. Websites stated, for example, that research in this area is “a rapidly growing field of biomedicine” [Clinique OVO], that “many new potential applications [are] being developed every year… the likelihood of potential use is increasing… the most exciting use of cord blood is yet to come” [Healthcord], and that “stem cells have been used in clinical trials to repair damaged tissues and organs (regenerative medicine), and the outcomes have been promising” [Progenics]. Two websites stated that “at some point in life” or “in the future,” “1 in 3 people” may benefit from “stem cell therapies” or “regenerative medicine” [Future Health, Insception]. CreATe’s website reads of stem cells’ “incredible potential” stating that “we are on the cusp of being able to use these stem cells lung cancer, breast cancer, heart disease, multiple sclerosis, lupus, AIDS, strokes, spinal cord damage, diabetes, cerebral palsy, kidney disease, and many other diseases.”

Regenerative and speculative stem cell therapies also had a presence in testimonials and client stories. As displayed in Table 1, three companies had testimonials appearing on their homepage, and five had testimonials or client stories somewhere on their website. Of these five, three companies detailed success stories using stem cells in regenerative therapies.

Cells for Life told the story of a daughter suffering from cerebral palsy who received a stem cell transplant from the cord blood of a newborn sibling. It read that after the infusion, the child “wouldn’t get tired anymore” and showed a “marked improvement” in speech, outcomes which “have exceeded expectations.” The family noted, “We want all new or expecting parents to be educated and be aware of the option to store cord blood.” CReATe’s homepage features a client story as covered by *The National Post* in 2016, which focused on a child who suffered from hypoxic ischemic encephalopathy at birth and was treated with stem cells from his banked cord blood. The post read that “within 24 h after the transplant, there was a remarkable clinical improvement in his condition” and that while he now has cerebral palsy, “his development has far surpassed doctors’ expectations.” In the video attached to the post, the boy’s mother says, “even though there is no hard medical evidence to what the stem cells have done, I know that they have done something, and to this day we are so grateful that we did it—because had we not done it, today we would be living with the biggest regret of our lives.” A regenerative success story for cerebral palsy was also presented on Future Health’s testimonial section. Some stories of regenerative success for cerebral palsy and autism are displayed in the blog section of Insception’s website.

##### Likelihood of use

Information around the likelihood that stored cord blood will be used by clients contained both explicit and implicit messaging. None of the websites’ homepages presented information on the likelihood of use, and three of the seven websites did not explicitly provide any information in this regard. Three websites belonging to Progenics, Cells for Life, and Insception all use the research paper of Neitfeld and Verter (2008) to state an estimated likelihood of use of 1/400. For example, Cells for Life’s website reads, “Some people suggest that the chance of using a cord blood sample is 1 in 10,000. Others estimate 1 in 2700. There is a recent publication by Neitfeld et al. (2008) that estimates that the chances of a person using a stored cord blood sample before the age of 70 is about 1 in 400 based on current treatments.” It then states, “When the addition of future treatment applications for diseases is discovered these odds of use will increase.” Insception’s website states, “With the ever-growing list of potential uses and research, the possibility of your family benefiting from a cord blood stem cell transplant also increases. Cord blood is an acceptable source of stem cells worldwide. Current research shows that there may be up to a 1:200 chance that you or a family member will benefit from a stem cell transplant in your lifetime^17^.” Reference 17 links to the 2008 publication by J.J. Nietfeld.

Implicit messaging on websites commonly detailed the number of transplants that have taken place worldwide (i.e. Insception 40,000+ , Cells for Life, 4000/year, etc.) which may or may not give the impression of how these absolute numbers of use are perceived. Additionally, many of the websites presented information around the number of clinical trials taking place, and a few websites mentioned the number of samples stored and that some clients’ samples have been used for treatments. Academic literature has discussed the impactful role that private banking might play in the development of research (Isasi et al. 2013). Few private banks, however, stressed this message on their websites. Indeed, the only website with rhetoric of this nature belonged to Insception, where the “opportunity to participate in emerging clinical trials” was mentioned as an “advantage” to family banking.

#### Theme of storing options

##### Portrayal of public banking

**Fig. 1:**
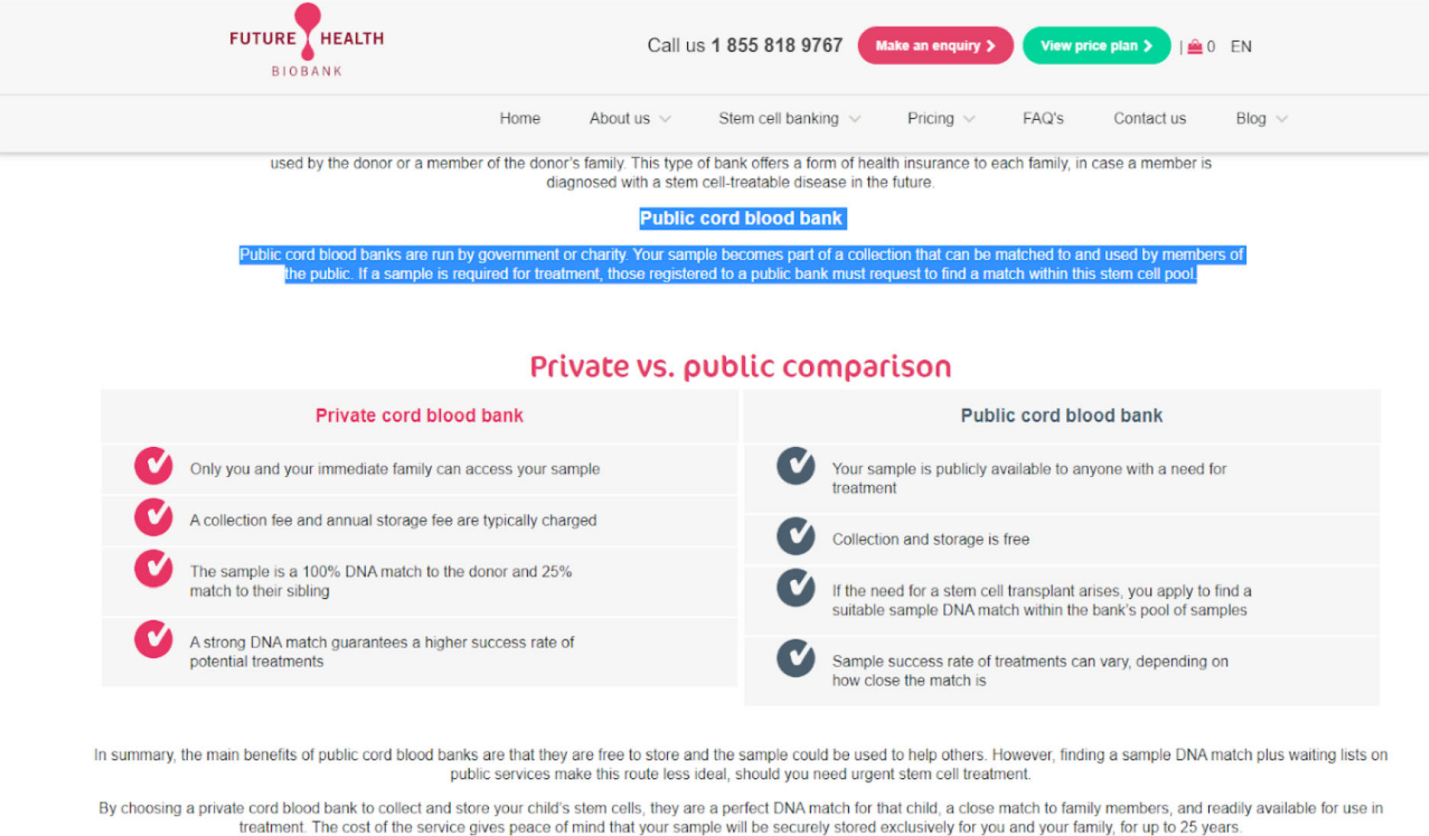
Screenshot of Future Health’s website showing chart comparing private versus public cord blood banking services

Information about public cord blood banking was presented on five of the seven websites, but the manner in which public banking was portrayed varied significantly. Healthcord and Progenics’ websites contained no information around public banking. Three websites, Future Health, Cells for Life, and Insception included specific text outlining the differences between public and private banking. See, for example, Fig. 1. Here, the common theme was to highlight the superiority of private storage as it offers a “perfect match” (Future Health), is preferred by physicians performing transplants (Cells for Life), is more easily-accessible (Cells for Life), and provides guaranteed access where a public bank cannot (Insception). Insception’s website also states that there are “limited options for public storage.” CReATe’s website detailed in videos how when banking publicly, individuals “lose their rights to the sample” whereas in private banks those rights are “retained…for your family.” CReATe’s website also details how the public criteria are more “strict,” resulting in a lower storage success rate.

##### Portrayal of storage versus waste

Common messaging across the websites suggested that cord blood is either stored privately or discarded. As previously mentioned, two websites, Progenics and Healthcord, made no mention of the public banking option. These two companies’ websites, in addition to Cells for Life, Insception, and CReATe, had some messaging that presented the discarding theme. Progenic’s website included the message that cord blood has “life-saving potential that is too precious to waste.” Healthcord stated that the storage option provides “access to a stem cell source” that “would otherwise be discarded as medical waste.” In CReATe’s FAQ it reads, “If the umbilical cord is discarded after your baby is delivered, the chance to preserve their precious stem cells is lost forever.”

#### Theme of social benefits (benefits aside from clinical treatments)

The stated social benefits around private cord blood banking touched on themes related to making a responsible family-based decision in order to ensure safety and potential success in the future. Here, cord banking was often described as insurance or as an investment that helps one prepare for the future while providing emotional comfort. Future Health’s website, for example, described stem cells as “a type of biological insurance” that could be used to save lives in the future. Clients deciding to store are “investing not only in their [own] future health, but also that of the rest of [their] family’s.” The testimonials section of Future Health’s website relayed a family’s experience as the following: “We have decided to use this approach with the hope that our children will never need to use stem cells, but it seems important to give them this extra option. The progress of medical research generally and in particular for stem cells is simply unbelievable and we don’t know what will be possible tomorrow.” Healthcord’s website analogized cord blood banking with the comforts of insurance as it stated that “the hope is that no child will ever need his or her cord blood, but cord blood banking offers peace-of-mind that your child’s cord blood is available if he or she needs it. Most people purchase home insurance every year despite the small odds that their home will ever burn down because they can’t afford to buy another house, so they cannot take the chance.” Likewise, testimonial videos on CReATe’s website had clients who say it is a “good investment in [their] family’s future,” that it provides “peace of mind” and that it is a “small investment that one day could yield a huge return.”

The rhetoric around cord blood banking as an “investment” or means of “insurance” also took on characteristics of responsible decision-making in the particular context of being or becoming parents. Healthcord’s website, for example, included the text that “Cord blood banking allows you to enjoy the security knowing that you have given your child a distinct health advantage in life.” On Clinique OVO’s website it read, **“**As parents we always want to be sure we do everything for our children to help them build a strong and healthy future. At OVO biosurance we truly believe that cord blood banking is an excellent opportunity to give your child the chance for a better future.” Other websites contained this messaging in testimonials. On Cells for Life’s website, for example, a testimonial read, “We took so long to get pregnant and have a baby and the last thing we wanted to do was put that child’s life in danger. If there’s anything you can do to protect the child, you’re going to do it.”

The last theme of the social benefits pertains to the idea of urgency based on a limited opportunity that must be seized in order to reap potential benefits. Progenics’ website stated that banking cord blood is a “once-in-a-lifetime” opportunity because stem cells “can only be collected at birth but the life saving [sic] potential of these valuable cells can last a life-time.” Clinique OVO’s website read that “the birth of a child is a unique opportunity to harvest the precious stem cells found in the umbilical cord blood cells.” CReATe’s website included the following, “If the umbilical cord is discarded… the chance to preserve their precious stem cells is lost forever…once that baby is born and that cord blood is thrown in the garbage… you will never have another chance to do this.”

## Discussion

The common marketing trait across all websites was that cord blood stem cells (and cord tissues) have high value for current and future use. Websites did not typically foreground the likelihood of use, and in cases where they did, they provided much higher odds than can be found on government websites (Shearer et al. 2017). Instead of foregrounding the probability of use, companies detaileded the high number of current stem cell applications (most commonly portrayed as treatments for 80+ diseases), and how cord blood stem cells are a “perfect” match for donors and possess potential for family use. Companies also highlighted the number of transplant procedures in which their samples had been used, highly successful or perfect transplant and storage rates, and testimonials and family stories detailing successful use. Issues have been raised in Canada, however, concerning quality of storage and ease of accessibility, which have resulted in litigation (CBC News 2020). It is potentially in light of cases like these that public attention has been given to promoting quality and trustworthiness.

The potential for future use of stem cells was strongly promoted on the websites, whereby the expected use was portrayed to increase dramatically. This promissory aspect of cord blood stem cell use (Brown 2013; Martin et al. 2008; Petersen and Krisjansen 2015) was detailed through explicit text, testimonials and family stories, and was often linked to a large number of clinical trials underway. Here, stem cells were labelled as “precious” and as having “enormous potential,” which could impact the lives of “1 in 3 people” in the future. The future use of stem cells was further enforced with the detailing of storage equipment that can preserve stem cells for “20 years,” “30 years” or “theoretically indefinitely,” therefore enabling ample time for the science to develop and for clients to potentially benefit. This language leverages the existing—and largely unfounded—hype surrounding stem cell research, a rhetorical strategy that is frequently used to market unproven therapies (Caulfield et al. 2016; Sipp et al. 2017). The marketing testimonials in particular, portraying positive and emotionally-charged anecdotes, might play a key role in hyping the benefits and in turn influencing customers’ decisions (Hawke et al. 2019).

Messaging was also evident on the websites suggesting that there are only two options for cord blood: to discard or to privately store. While the option to donate cord blood was included on some websites, it was, unsurprisingly, portrayed as an inferior option to private storage. In addition to the discard vs. bank messaging was the rhetoric around “investing” in cord blood, and taking “peace of mind” in the “insurance” it provides. This messaging has the potential to greatly increase pressure on parents to make wise, educated decisions for their future children. Indeed, testimonials and family stories mentioned these aspects in their own decision making, in some cases highlighting the regret they might have felt if they had decided not to store. Narrative messaging of this sort can have a powerful impact on families making health-related decisions (Caulfield et al. 2019; Fagerlin et al. 2005; Hawke et al. 2019), a dynamic which is likely intensified in the stressful period of pregnancy and child bearing when women and families can experience an overwhelming influx of information.

Questions should be asked about whether the promissory aspects of these websites constitute breaches of Canadian law or regulation. For example, research has shown that the maternal blood draw can be a complex and arduous process for women who have recently given birth (Haw 2016) and yet the maternal blood draw is seldom made clear and transparent on many websites. The larger issue, however, concerns the portrayal of speculative future regenerative therapies, particularly in regard to autism, and whether these marketing materials could breach advertising law. Because the problematic marketing of private cord blood banking for future use often relates to speculative future cell therapies that do not exist and are not being advertised for current clinical use, most private blood bank marketing seems to fall outside Health Canada’s regulatory scope, which is generally limited to health products and devices. Yet, as we have argued in a separate legal analysis, claims about speculative future treatments that are misleading in a way that can influence a consumer’s behaviour or purchasing decisions are illegal in Canada under the *Competition Act* (Murdoch et al. 2020). Moreover, the legal standard by which the general impression of an advertisement is judged likely assumes that consumers are “credulous and inexperienced” (Richard v. Time Inc., access 2020). This means that in Canada, there is a strong obligation for advertisers to prevent unwitting consumers from being misled. Since all the potential benefit of private banking is derived from its potential future use for treatment, we can certainly say that unsupported claims that misrepresent or exaggerate the present or future potential of cord blood-related treatments are materially misleading and likely to affect consumer behaviour. The noted statistical claims on private bank websites about likelihood of usage are also potentially misleading, as they seem to diverge from the more widely accepted and much lower probabilities found in public sources and elsewhere. These claims could be other breaches of advertising law.

## Conclusion

The online marketing of private cord blood banking on company websites is concerning. There was substantial hype evident on the websites, amplifying the promise of speculative uses and distorting the likelihood of use. The promotion of cord blood banking as a measure of insurance taken by responsible parents looking to do the best for their newborn children will likely resonate on an emotional level for some, perhaps leading to decisions being made with inaccurate expectations. With cord blood banking expected to grow in Canada and around the world, careful monitoring of these business practices is important to ensure that the Canadian public is adequately and accurately informed of the services being offered.

## Data Availability

Data can be made available upon reasonable request.
